# Whole-genome sequencing of the endangered bovine species Gayal (*Bos frontalis*) provides new insights into its genetic features

**DOI:** 10.1038/srep19787

**Published:** 2016-01-25

**Authors:** Chugang Mei, Hongcheng Wang, Wenjuan Zhu, Hongbao Wang, Gong Cheng, Kaixing Qu, Xuanmin Guang, Anning Li, Chunping Zhao, Wucai Yang, Chongzhi Wang, Yaping Xin, Linsen Zan

**Affiliations:** 1 College of Animal Science and Technology, Northwest A&F University, Yangling Shaanxi, China; 2 BGI-Tech, BGI-Shenzhen, Shenzhen, China; 3 Yunnan Academy of Grassland and Animal Science, Xiaoshao Kunming, Yunnan, China

## Abstract

Gayal (*Bos frontalis*) is a semi-wild and endangered bovine species that differs from domestic cattle (*Bos taurus* and *Bos indicus*), and its genetic background remains unclear. Here, we performed whole-genome sequencing of one Gayal for the first time, with one Red Angus cattle and one Japanese Black cattle as controls. In total, 97.8 Gb of sequencing reads were generated with an average 11.78-fold depth and >98.44% coverage of the reference sequence (UMD3.1). Numerous different variations were identified, 62.24% of the total single nucleotide polymorphisms (SNPs) detected in Gayal were novel, and 16,901 breed-specific nonsynonymous SNPs (BS-nsSNPs) that might be associated with traits of interest in Gayal were further investigated. Moreover, the demographic history of bovine species was first analyzed, and two population expansions and two population bottlenecks were identified. The obvious differences among their population sizes supported that Gayal was not *B. taurus*. The phylogenic analysis suggested that Gayal was a hybrid descendant from crossing of male wild gaur and female domestic cattle. These discoveries will provide valuable genomic information regarding potential genomic markers that could predict traits of interest for breeding programs of these cattle breeds and may assist relevant departments with future conservation and utilization of Gayal.

Gayal (*Bos frontalis*), also known as mithan or mithun, is a unique semi-wild and endangered bovine species that is distributed in hilly areas of China, India, Bangladesh, Myanmar and Bhutan^1^. The chromosome number of Gayal (2n = 58) differs from those of domesticated cattle (*Bos indicus* and *Bos taurus*, 2n = 60) and gaur (*Bos gaurus*, 2n = 56)^2^,^3^,^4^. Thus far, the origin of Gayal remains unclear. Some researchers presumed that Gayal was an independent species^5^,^6^,^7^, and some thought that it was a hybrid descendant from crossing of wild gaur and domestic cattle^8^,^9^. Previous reports have shown that Gayal can interbreed with domestic cattle (*B. taurus* and *B. indicus*), and that the female offspring may be fertile, whereas the male offspring may not always be fertile^10^,^11^,^12^. The phenotypic characteristics of Gayals have been retained under natural selection over time and have not been affected by artificial selection. This animal has a bulging forehead ridge above the nose, a pair of wide and short ears and feet with white stockings that can be used to distinguish them from domesticated cattle^2^,^13^. Gayals usually eat any variety of plant, including tree and bamboo leaves, grasses, reeds, etc. and have wide range of adaptations under harsh conditions, and salt is one of the favorite food items of this animal^14^,^15^. Moreover, Gayals have better performance in body size and meat quality than certain indigenous domestic cattle and can be used to breed hybrids to produce better meat and milk traits^11^,^16^.

Because the population size of Gayal is dwindling rapidly, they have been classified as an endangered species by the International Union for Conservation of Nature and Natural Resources (www.iucnredlist.org/). However, the specific inherited characteristics of Gayals are far from comprehensive, and their potential value has yet to be discovered. Thus, tapping the genetic information of this endangered semi-wild bovine species requires a timely and effective approach, especially considering the value and importance of this genetic resource.

Since the bovine genome and HapMap projects have been completed^17^,^18^,^19^, the whole-genome resequencing of multiple cattle breeds has progressed rapidly, including *B. taurus* (Hereford, Angus, Hanwoo, Yanbian, and Japanese native cattle), *B. indicus* (Gir, Nellore), dairy cattle (Holstein, Fleckvieh) and yak^20^,^21^,^22^,^23^,^24^,^25^,^26^,^27^. With the rapid development of science and technology, whole-genome sequencing has become one of the most important and effective methods for exploring the genetic information of different species.

However, the whole-genome sequencing of Gayal has not been performed. In this study, we sequenced the whole genome of one Gayal from Yunnan Province, China using an Illumina HiSeq 2000 and sequenced one Red Angus (RAN) and one Japanese Black cattle (JBC) as controls because both are breeds of the globally popular species *B. taurus*. A comparative diversity study may lead to a whole-genome analysis of the genetic features of Gayal, increase the understanding of the correlation between their phenotypic characteristics and genetic features and assist relevant departments with their conservation.

## Results and Discussion

### Sequencing and mapping

Whole-genome sequencing of one Gayal as well as one RAN and one JBC (Table S1) was performed on a HiSeq 2000 using genomic DNA, and 97.8 Gb of high quality paired-end reads (100 bp) was generated. To the best of our knowledge, this study is the first to perform whole-genome sequencing of Gayal. All of the obtained reads were mapped to the reference genome UMD3.1^19^ using BWA^28^. The reference sequence was 98.44% covered by the reads for Gayal and presented a 13.06-fold depth. These values are not significantly lower than are those of RAN (98.65%, 11.94-fold) and JBC (98.66%, 10.34-fold) despite the lower chromosome number in Gayal compared with that of *B. taurus* (Table 1). We argue that this phenomenon is a result of centric fusion involving ROB (2; 28), which has been reported by previous studies^2^,^3^, rather than chromosome deletion. Furthermore, the depth and coverage are sufficient to detect high-quality variations compared with previous sequencing studies in cattle^20^,^21^,^22^.

### Single nucleotide polymorphisms (SNPs) detection

Through comparisons with the reference genome, 28,493,996 SNPs (Table 2) were detected in these three sequenced cattle genomes. Of these SNPs, 53.23% (15,167,354 SNPs) were novel compared with the latest version of the cattle SNP database (dbSNP Build 140; ftp://ftp.ncbi.nlm.nih.gov/snp/organisms/cow_9913/chr_rpts/). As expected, the total variation number and novel rate in Gayal (23,828,562 SNPs, 62.24% novel) were all much higher than those in RAN (5,785,690 SNPs, 2.53% novel) and JBC (5,956,686 SNPs, 5.10% novel) because of differences between the genome sequences of *B. frontalis* and *B. taurus* and because of the semi-wild nature of Gayal compared with the domesticated nature of *B. taurus*. SNP annotation showed that 78.2% of the SNPs were located in intergenic regions (Gayal: 77.9%; RAN: 79.5%; JBC: 79.4%); 21.1% were located in genic regions, including intronic regions, splicing sites, exonic regions and untranslated regions; and the remaining 0.7% were located in up/downstream regions. The number of Gayal SNPs is as proportional to the length of chromosomes as those of RAN and JBC (Figure S1), which also supports the hypothesis that the two fewer chromosomes in Gayal compared with *B. taurus* resulted from a centric fusion involving ROB (2; 28).

Transition-to-transversion (TS/TV) ratios were calculated as indicators of potential random sequence errors^22^. Here, the TS/TV ratios (Gayal: 2.32, RAN: 2.17, JBC: 2.18) approximated the empirical human TS/TV ratio >2.1, indicating the high quality of the identified SNPs in an oblique manner. Furthermore, the homozygous/heterozygous ratios of Gayal, RAN and JBC were 1:0.8, 1:1.5 and 1:1.1, respectively. The finding that the ratio of Gayal was higher than were those of the other two breeds was somewhat surprising, because Gayal has been regarded as indigenous with its population size decreasing rapidly over recent thousands of years, which might have been fueled by natural directional selection on genotype.

### Insertions/deletions (InDels), copy number variations (CNVs) and structure variations (SVs) detection

**Figure 1:**
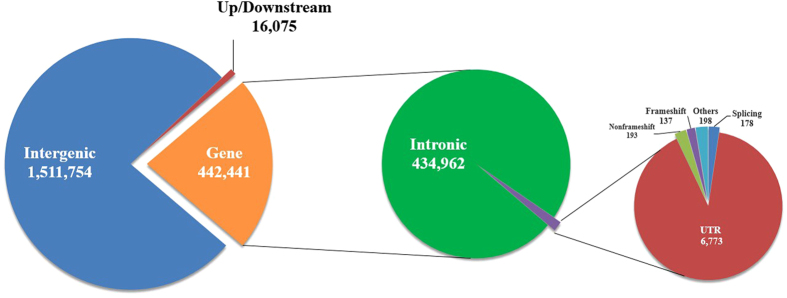
Identified InDels in Gayal. Variations were annotated using the RefSeq and Ensembl gene sets. Of the total InDels, 1,511,754/76.7% were located in intergenic regions; 442,441/22.5% were located in genic regions, including intronic, splicing sites, exonic and untranslated regions; and the remaining 16,075/0.8% were located in up/downstream regions.

In the current study, we found 2,352,519 InDels (Gayal: 1,970,270; RAN: 503,187; JBC: 461,234) (Table 3), with 55.78% (Gayal: 64.50%; RAN: 9.52%; JBC: 10.45%) being new. The distribution of the InDels is shown in Figure S1. The length of most InDels was 1 bp (Figure S2). Of the total InDels, 1,814,875/77.1% were located in intergenic regions (Gayal: 1,511,754/76.7%; RAN: 396,418/78.8%; JBC: 362,585/78.6%); 518,564/22.0% were located in genic regions (Gayal: 442,441/22.5%; RAN: 102,841/20.4%; JBC: 95,186/20.6%), including intronic, splicing sites, and exonic; and the remaining 19,080/0.9% were located in up/downstream regions (Table 3, Fig. 1).

In addition, large numbers of CNVs and SVs were identified in Gayal (3,659 CNVs and 70,810 SVs), RAN (2,607 CNVs and 49,890 SVs) and JBC (2,925 CNVs and 39,482 SVs) (Table S2).

As shown, InDels and SNPs were far more frequent in Gayal than in the other two breeds revealing that Gayal retained greater genetic diversity. The high proportions of novel SNPs and InDels found in Gayal suggest their potential value.

### SNP annotation and functional enrichment analysis of breed-specific nonsynonymous SNPs in Gayal

Variations were annotated using the RefSeq and Ensembl gene sets. Of the total SNPs in Gayal, 4,947,017 were in introns, 166 were in splice-sites, and 142,983 were in exonic regions, including 66,866 in untranslated regions (UTRs), 20,499 nonsynonymous, 47,996 synonymous, 62 stop-gain and 20 stop-loss (Table 2).

Genome-wide panels of SNPs have recently been used for predicting complex traits of plant and animal species^29^,^30^; Nonsynonymous SNPs (nsSNPs), leading to an amino acid change in the protein product, comprise a group of SNPs that are of particular interest, these SNPs are believed to have strong impact on phenotype. We further performed functional enrichment analysis of the genes including the breed-specific nsSNP (BS-nsSNP) sets in this study. These BS-nsSNPs have no overlap with that of other two breeds, and may reflect the breed-characteristics.

By comparison with RAN and JBC, we identified 16,901 BS-nsSNPs among 6,167 genes in Gayal (Figure S3). 2,842 of those genes were successfully aligned to the Cattle QTL database (http://www.animalgenome.org/cgi-bin/QTLdb/BT/index) (Table S3). In addition, we performed functional enrichment analysis on the using Database for Annotation Visualization and Integrated Discovery (DAVID) tools^31^,^32^. KEGG enrichment analysis results showed that 30 genes were significantly enriched in the complement and coagulation cascade pathway (ID: bta04610, P = 6.95 × 10^−12^) after Bonferroni correction (Table S4, Figure S4). The complement and coagulation cascade is a complex innate immune surveillance system that plays important roles in host homeostasis, inflammation, and pathogen defense^33^. According to previous studies, the C3AR1 gene (Table S4) isinvolved in protecting myeloid and lymphoid cells against *Listeria monocytogenes* induced apoptosis^34^. The MBL2 gene is associated with upper respiratory tract infection^35^, and A2M and BDKRB1 genes are involved in the immune response^36^,^37^.

In addition, GO analysis showed that after Bonferroni correction, the BS-nsSNP containing genes were significantly enriched in terms including carbohydrate binding (GO: 0030246, P = 1.75 × 10^−8^), polysaccharide binding (GO: 0030247, P = 1.12 × 10^−6^), and glycosaminoglycan binding (GO:0005539, P = 3.43 × 10^−6^). Besides, these genes were also significantly enriched in terms associated with environmental adaptations, such as biological adhesion (GO: 0022610, P = 7.82 × 10^−6^), regulation of response to external stimulus (GO: 0032101, P = 8.02 × 10^−6^), defense response (GO: 0006952, P = 1.07 × 10^−5^) and peptidase activity (GO: 0008233, P = 4.78 × 10^−5^) (Table S4).

Recent studies have shown signatures that mutations in some of these enriched genes have potentially affect compex traits in cattle. For example, nsSNPs in MBL2 and LBP gene were significantly correlated with mastitis in cow^38^,^39^, and MBL2 haplotypes influenced *B. abortus* infection in the water buffalo (*Bubalus bubalis*)^40^. Polymorphisms in Toll-like receptor genes (TLRs) were closely related to innate immunity in farm animals^41^, and some nsSNPs found in the bovine Toll-like receptors TLR1, TLR2 and TLR4 gene were confirmed to have association with natural resistance to *Mycobacterium avium subsp. paratuberculosis* infection in cattle^42^,^43^. Besides, previous reports suggested that nsSNPs in Cathelicidins (CATHLs) have with respect to bovine innate immunity^44^, too.

Thus, we speculate that these Gayal-special nsSNP sets can be useful genomic resources to further test how these genes are genetically implicated with characteristics of Gayal.

### Other functional analyses

#### SNP annotation and functional enrichment analysis of breed-specific nsSNPs (BS-nsSNPs) in RAN and JBC

Similarly, 1,892 and 1,936 BS-nsSNPs detected in the RAN and JBC genomes were investigated, respectively. In total, 669 of 1,410 BS-nsSNP containing genes in RAN were annotated in cattle QTL regions, and as were 654 of 1,409 BS-nsSNP containing genes in JBC (Figure S3, Tables S5 and S6).

#### InDels annotation and functional enrichment analysis of breed-specific InDels (BS-InDels) in sequenced cattle

As presently research, we also focused on loss-of-function (LOF) InDels (stop-gains, frameshift InDels in the coding sequence and disruptions to essential splice sites) that may lead to functional changes in the genes in which they are located in^45^. In total, 160, 38 and 33 breed specific LOF-InDels were obtained in Gayal, RAN and JBC, and 66/142, 12/34 and 14/33 BS-LOF-InDel containing genes were detected in cattle QTL regions, respectively (Figure S5, Tables S7, S8 and S9).

However, functional enrichment analysis of breed-specific nsSNPs (BS-nsSNPs) in RAN and JBC and of BS-LOF-InDel containing genes in Gayal, RAN and JBC showed that these genes were not significantly enriched in any term after Bonferroni correction.

The functional enrichment analyses indicated potential correlations between the phenotypic characteristics and genetic features of the Gayal breed. Nevertheless, population genetic studies in this subspecies are required to validate the identified BS-nsSNPs and to examine their association with relevant phenotypic traits. Additionally, most BS-nsSNPs that have been identified in Gayal have yet to be reported, and their functions and associations require investigation. However, the new BS-nsSNPs identified in this study will be valuable for future research.

### Demographic history

**Figure 2:**
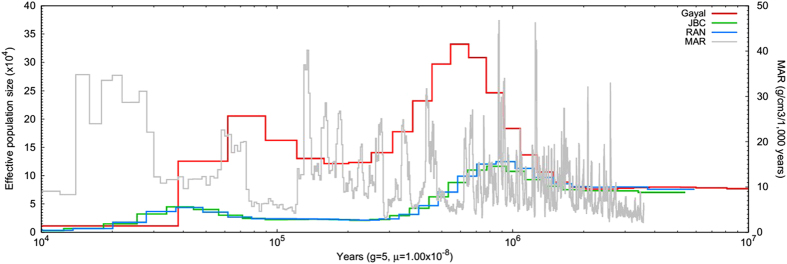
Demographic history of Gayal (*Bos frontalis*), JBC and RAN (*Bos taurus*). Autosomal SNPs of gayal were used to reconstruct demographic history with the pairwise sequentially Markovian coalescent (PSMC) model with the generation time (g = 5), mutation rates (μ = 1 × 10^−8^). As is showed above, both of *B. frontalis* (Gayal) and *B. taurus* (RAN and JBC) have undergone two population expansions and two population bottlenecks, and gayal could be clearly distinguished from RAN and JBC, which strongly supported that gayal was not a *Bos taurus*.

The pairwise sequentially Markovian coalescent (PSMC) model^46^ was used to reconstruct the demographic history of *B. frontalis* and *B. taurus*. The demographic history of Gayal showed population peaks at ~0.6 million years ago (Mya) and ~80 thousand years ago (Kya) and population bottlenecks at ~0.2 Mya and ~40 Kya (Fig. 2 and Figure S6). Notably, these fluctuations in effective population size (Ne) were significantly negatively correlated with changes in the amount of atmospheric dust, as inferred by the mass accumulation rate (MAR) of Chinese loess^47^ (Pearson’s correlation R = −0.35, P < 0.05), an index indicating cold and dry or warm and wet climatic periods in China. This result was similar to that of a previous study^48^.

The two population bottlenecks of *B. frontalis* and *B. taurus* occurred during the penultimate glaciation and last glaciation^49^, respectively, which may have led to the degeneration of grasslands and the establishment of forests. In contrast, the two population expansions occurred during first interglaciation and after the retreat of the penultimate glaciation^49^, respectively, which indicate warm and wet weather conditions that provided sufficient moisture and suitable temperatures to facilitate the lush growth of grassland vegetation and population expansion.

The *B. taurus* (RAN and JBC) populations also correlated with climatic or environmental changes (Fig. 2, Figures S6 and S7). However, differences were observed between *B. frontalis* and *B. taurus*, including differences in their effective population size and population decline or expansion rate. The divergent population histories strongly suggest that *B. frontalis* is not a *B. taurus*. Population size of Gayal decreased later during glaciation and expanded earlier (compared to *B. taurus*) when climate became favorable. This finding potentially supports the view that *B. frontalis* better tolerances extreme environmental changes compared with *B. taurus*^14^,^15^,^50^.

### Phylogeny of bovine-related species

To explore the genetic relationships between Gayal (*B. frontalis*) and other Bovinae subfamily members, we performed phylogenetic analysis using available data. Twenty randomly selected single ortholog copy genes in *B. taurus*, *Bos mutus* (wild yak) and *Bubalus bubalis* genome and the completed mitochondrial genomes of *B. taurus*, *B. indicus*, *B. gaurus*, *B. mutus* (wild yak) and *Bubalus bubalis* were download from NCBI. *Equus caballus* was used as an outgroup (Tables S10 and S11).

**Figure 3:**
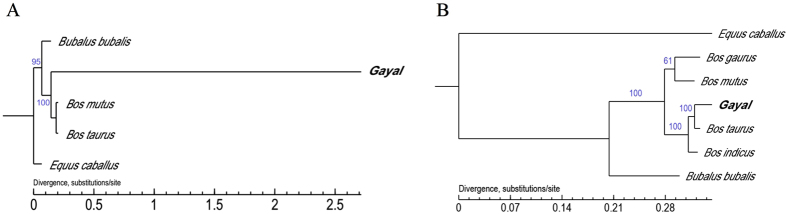
Phylogeny of bovine-related species. Phylogenetic analysis using available data, including (**A**) 20 randomly selected single ortholog copy genes in *Bos taurus*, *Bos mutus* (wild yak) and *Bubalus bubalis* genomes and **(B)** the completed mitochondrial genomes of *Bos taurus*, *Bos indicus*, *Bos gaurus*, *Bos mutus* (wild yak) and *Bubalus bubalis* downloaded from NCBI, and *Equus caballus* was used as an outgroup.

The maximum likelihood tree based on single ortholog copy genes (Fig. 3A) places Gayal (*B. frontalis*) off *B. mutus* and *B. taurus* also indicating that Gayal is clearly distinct from *B. taurus*. The maximum likelihood tree based on the mitochondrial genomes (Fig. 3B) shows that Gayal is the nearest species to domesticated cattle (*B. taurus* and *B. indicus*), implying that Gayal (*B. frontalis*) is a hybrid descendant from crossing of male wild gaur and female domestic cattle. This case is supported by the results presented by other reports^8^,^9^.

## Conclusions

In the present study, whole-genome sequencing with next-generation sequencing technology was performed for the endangered bovine species Gayal (*B. frontalis*) for the first time, and the results were compared with those of two ubiquitous breeds of domesticated beef cattle: RAN and JBC. A large number of genetic variations were identified, including SNPs, InDels, CNVs and SVs. Sequencing coverage and variation distribution support the hypothesis that the two fewer chromosomes in Gayal compared with those of the other bovine species was a result of chromosome fusion^2^,^3^. The genetic variation annotations showed that more than half of the SNPs or InDels in Gayal were new and that significantly more variations were found than in RAN and JBC, indicating that Gayal is a valuable potential genetic resource that has not yet been exploited. Compared with RAN and JBC, the functional annotation of the breed-specific-SNP/InDel containing genes in Gayal showed a potential correlation between its phenotypic characteristics and genetic features. Moreover, demographic history analysis of cattle was reported in this study for the first time. The results of this analysis showed that all the three cattle breeds have undergone two population expansions and two population bottlenecks, and Gayal (*B. frontalis*) could be clearly distinguished from RAN and JBC (*B. taurus*). This observation strongly supports the contention that Gayal is not *B. taurus* in lineage. In addition, the phylogenic analysis based on single ortholog copy genes places Gayal (*B. frontalis*) as phylogenetically distinct from both *B. mutus* and *B. taurus*, while phylogenic analysis based on mitochondrial genomes shows that Gayal is closely related to domesticated cattle (*B. taurus* and *B. indicus*). Thus, reasonable conjecture based upon these observations is that Gayal is a hybrid descendant from crossing of male wild gaur and female domestic cattle.

In conclusion, our study is the first whole-genome sequencing of the endangered bovine species Gayal (*Bos frontalis*) that provides new insights into its genetic features. The results could predict traits of interest for further cattle breeding programs and may assist relevant departments with future conservation and utilization of Gayal.

## Methods

### Samples preparation and sequencing

For this experiment, we collected blood samples of one Gayal from Yunnan Province, China, as well as one Red Angus (RAN) from Xinjiang Province China, and one Japanese Black cattle (JBC) from Anhui Province, China (Table S1). The experiments and animal care were performed according to the Regulations for the Administration of Affairs Concerning Experimental Animals (Ministry of Science and Technology, China, 2004) and approved by the Institutional Animal Care and Use Committee (College of Animal Science and Technology, Northwest A&F University, China). For each individual, at least 5 μg of genomic DNA extracted from the blood was sheared into fragments of 200–800 bp using the Covaris system (Life Technologies). DNA fragments were then end repaired, A-tailed, ligated to paired-end adaptors and PCR amplified with 500-bp inserts for library construction. Sequencing was performed on the HiSeq 2000 platform (Illumina), and100-bp pair-end reads were generated.

### Alignment and variation identification

Pair-end reads (100 bp) obtained from sequencing were mapped to the reference UMD3.1^19^ using BWA software^28^. The detailed parameters were as follows: ‘bwa aln –m 200000 –o 1 –e 30 –i 15 –L –I –t 4 –n 0.04 –R 20 -f’,‘bwa sampe –a 650 –n 30 –N 30’. After the alignment, the result of the SAM format file was converted to bam format using SAMtools^51^. The bam files were sorted and the duplicated reads were filtered based on the Picard pack. SNP and InDel calling was performed using the Genome Analysis Toolkit (GATK, version 2.4-9)^52^. Then, to obtain get high quality variants of Gayal, strict filter conditions were performed by GATK^53^ (DP < 4, DP > 60, MQ0 ≥ 4 && ($MQ0/(1.0*$DP)) > 0.1, QD < 2.0, FS > 200, QUAL < 30, MQ < 40, FS > 60, MQRankSum <−12.5, ReadPosRankSum <−8.0, and cluster parameters (-cluster 3 -window 10)). For SNPs, if HRun was more than 6, these SNPs were removed. For InDels, if HRun was more than 10, these InDels were removed. CNVs and SVs were identified using CNVnator v0.2.7^54^ and Breakdancer v1.2^55^, respectively, with default parameters.

### Variation annotation

The package ANNOVAR^56^ was used to identify whether variants caused protein coding changes and to identify the amino acids affected. ‘Upstream’ refers to a variant that overlaps with the 1 kb region upstream of the gene start site. ‘Stop gain’ means that a nonsynonymous SNP leads to the creation of a stop codon at the variant site. ‘Stop loss’ means that a nonsynonymous SNP leads to the elimination of a stop codon at the variant site. ‘Unknown’ means unknown function (due to various errors in the gene structure definition in the database file). ‘Splicing’ means that a variant is within 2 bp of a splice junction. ‘Downstream’ means that a variant overlaps with the 1 kb region downstream of the gene end site. ‘Upstream/Downstream’ means that a variant is located in downstream and upstream regions (possibly for two different genes). SIFT algorithm^57^ was used to predict the functional impact of the missense mutations. Variants were classified as ‘known’ if the non-reference allele was present in the dbSNP database and as ‘novel’ otherwise. The source databases used by Annovar during annotation included dbSNP Build 140 (ftp://ftp.ncbi.nlm.nih.gov/snp/organisms/cow_9913/chr_rpts/), Ensembl release 78, NCBI RNA RefSeq. The Ensembl gene and RefSeq gene set are available from the UCSC download site (ftp://hgdownload.cse.ucsc.edu/goldenPath/bosTau6/database).

### Functional enrichment analysis

We performed a functional enrichment analysis of genes that were found to have breed-specific nonsynonymous SNPs (BS-nsSNPs) or loss-of-function (LOF) InDels (stop-gains, frameshift InDels in the coding sequence and disruptions to essential splice sites) using DAVID tools^31^,^32^. More particularly, for Gayal, we chose the genes that contained at least three BS-nsSNPs in consideration of its extremely large count of mutations. To obtain get reliable results, Bonferroni correction was applied in all the enrichment analyses.

### Demographic history

Autosomal SNPs of the three sequenced cattle breeds were used to reconstruct demographic history with the PSMC model^46^ with the generation time (g = 5) and mutation rates (μ = 1 × 10^−8^). Parameters were set as follows: −N 30, −t 15, −r 5 −p ‘4 + 25*2 + 4 + 6’. Following Li’s procedure, we applied a bootstrapping approach, repeating sampling 100 times to estimate the variance of simulated results.

### Phylogeny of bovine-related species

To understand the genetic relationships between Gayal (*Bos frotanlis*) and other Bovinae subfamilies, we performed phylogenetic analyses by using available data, including 20 randomly selected single ortholog copy genes in *Bos taurus*, *Bos mutus* (wild yak) and *Bubalus bubalis* genome and the completed mitochondrial genomes of *Bos taurus*, *Bos indicus*, *Bos gaurus*, *Bos mutus* (wild yak) and *Bubalus bubalis* downloaded from NCBI. *Equus caballus* was used as an utgroup (Tables S10 and S11). The downloaded data were aligned by PRANK based on each gene name, and then the super gene was constructed as species by each gene in each species. Notably for Gayal, we mapped the obtained reads to *Bos taurus* mitochondria genome using SOAP, and generated a consensus sequence that was used as the super gene of Gayal. At last, we used PHYML to build phylogenetic trees based on GTR + gamma model for mitochondrial genomes and WGA model for single ortholog copy genes, respectively.

## Additional Information

**Accession Codes**:The sequencing reads have been deposited in the NCBI Sequence Read Archive (SRA) under accession SRA291190.

**How to cite this article**: Mei C.G. *et al*. Whole-genome sequencing of the endangered bovine species Gayal (*Bos frontalis*) provides new insights into its genetic features. *Sci. Rep.* **6**, 19787; doi: 10.1038/srep19787 (2016).

## Supplementary Material

Supplementary Information

Supplementary Table S3

Supplementary Table S4

Supplementary Table S5

Supplementary Table S6

Supplementary Table S7

Supplementary Table S8

Supplementary Table S9

Supplementary Table S10

Supplementary Table S11

## Figures and Tables

**Table 1:** Summary of sequence read alignments to the reference genome.

Summary	*Bos frontalis*	*Bos taurus*	
	Gayal	RAN	JBC
Mean depth	13.06	11.94	10.34
Mismatch rate	1.22%	0.48%	0.57%
GC content rate	41.82%	41.84%	41.92%
Coverage rate	98.44%	98.65%	98.71%
Coverage rate (>=1X)	98.37%	98.62%	98.66%
Coverage rate (>=4X)	96.43%	96.76%	95.15%
Coverage rate (>=8X)	85.92%	81.68%	69.94%
Coverage rate (>=12X)	57.47%	45.20%	28.56%
Total reads	362606690 (100%)	333277352 (100%)	282866734 (100%)
Mapped reads	357292180 (98.53%)	330198207 (99.08%)	280354187 (99.11%)
Reads that are properly paired	340787139 (93.98%)	320590349 (96.19%)	272111559 (96.20%)
Only one of the reads are mapped	3690443 (1.02%)	2248862 (0.67%)	1940525 (0.69%)
Reads with mate mapped to a different chromosomes	5314158 (1.47%)	3078939 (0.92%)	2512311 (0.89%)
unmapped reads	5314510 (1.47%)	3079145 (0.92%)	2512547 (0.89%)

**Table 2:** Functional classification of the detected single nucleotide polymorphisms (SNPs).

SNP	Gayal	RAN	JBC	Total
Total numbers	23,828,562	5,785,690	5,956,686	28,493,996
Transition/transversion ratio	2.32	2.17	2.18	2.27
Novel rate (%)	62.24%	2.53%	5.10%	53.23%
Intergenic	18,565,897	4,601,652	4,731,811	22,276,335
Upstream	80,850	18,804	18,623	96,119
Downstream	91,649	20,039	20,967	108,248
Gene	5,090,166	1,145,195	1,185,285	6,013,294
Intronic	4,947,017	1,111,361	1,150,941	5,842,593
Splicing	166	103	96	79,507
Exonic	142,983	33,731	34,248	91,194
UTR	66,866	15,682	15,629	208
Non-synonymous	20,499	5,652	5,897	25,401
Synonymous	47,996	10,253	10,514	56,278
Stopgain	62	30	26	93
Stoploss	20	8	5	22
Others	7,540	2,106	2,177	9,192

**Table 3:** Functional classification of the detected insertions/deletions (InDels).

InDels	Gayal	RAN	JBC	Total
Total numbers	1,970,270	503,187	461,234	2,352,519
Insertion	932,926	248,943	227,728	1,145,852
Deletion	1,037,344	254,244	233,506	1,206,667
Novel rate	64.50%	9.52%	10.45%	55.78%
Intergenic	1,511,754	396,418	362,585	1,814,875
Upstream	6,927	1,827	1,563	8,327
Downstream	9,148	2,101	1,900	10,753
Gene	442,441	102,841	95,186	518,564
Intronic	434,962	100,678	93,304	509,662
Splicing	178	135	120	245
Exonic	7,301	2,028	1,762	8,655
UTR	6,773	1,732	1,504	8,018
Nonframeshift	193	43	40	228
Frameshift	143	75	65	172
Others	192	178	153	237
