# Supplementary material for: Whole-genome sequencing of the endangered bovine species Gayal (Bos frontalis) provides new insights into its genetic features
Source: Sci Rep. 2016 Jan 25;6:19787. doi: 10.1038/srep19787 (PMC4726396; doi:10.1038/srep19787)
Supplement: Supplementary Information [file srep19787-s1.doc]

**Whole-genome sequencing of the endangered bovine species gayal (*Bos frontalis*) provides new insights into its genetic features**

Chugang Mei^1,4^, Hongcheng Wang^1,4^, Wenjuan Zhu^2,4^, Hongbao Wang^1^, Gong Cheng^1^, Kaixing Qu^3^, Xuanmin Guang^2^, Anning Li^1^, Chunping Zhao^1^, Wucai Yang^1^, Chongzhi Wang^2^, Yaping Xin^1^, Linsen Zan^1*^

^1^ College of Animal Science and Technology, Northwest A&F University, Yangling Shaanxi, China.

^2^ BGI-Tech, BGI-Shenzhen, Shenzhen, China.

^3^ Yunnan Academy of Grassland and Animal Science, Xiaoshao Kunming, Yunnan, China.

^4^ These authors contributed equally to this work.

^*^ Corresponding author.

Tel.: +86-29-87091923

Fax: +86-29-87092164

**E-mail:** [zanlinsen@163.com](mailto:zanlinsen@163.com)

**On-line Supplementary Data**

**Supplementary Figures:**


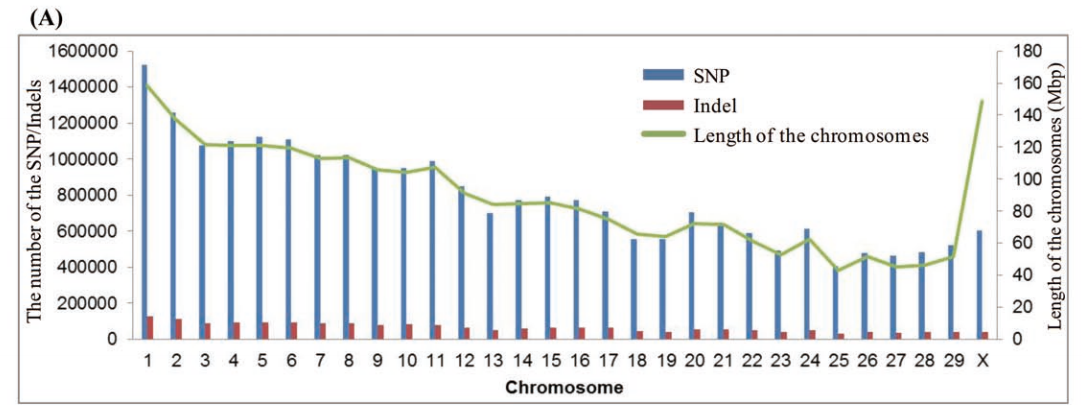


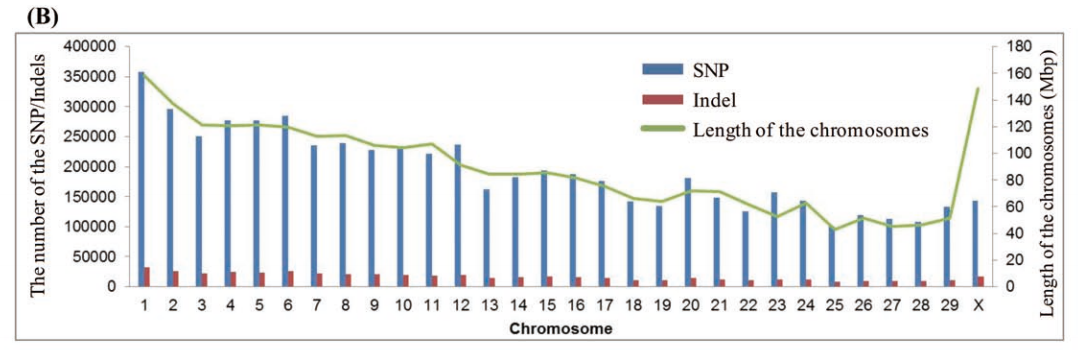


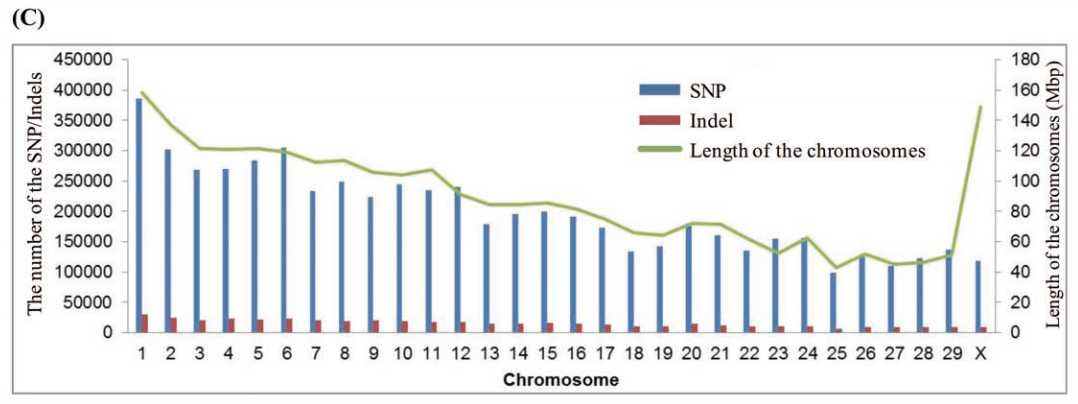


**Figure S1. Chromosomal distribution of the SNPs and InDels in (A) Gayal, (B) Red Angus cattle (RAN) and (C) Japanese black cattle (JBC).** The number of gayal SNPs is as proportional to the length of chromosomes as that of RAN and JBC, which supports the hypothesis that the two fewer chromosomes in gayal compared with *Bos taurus* resulted from a centric fusion involving ROB (2; 28).


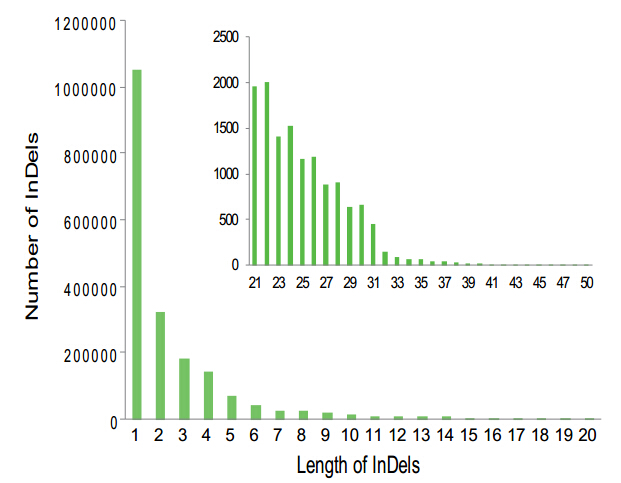


**Figure S2. Length distribution of the InDels identified.** The length of most InDels was 1 bp.


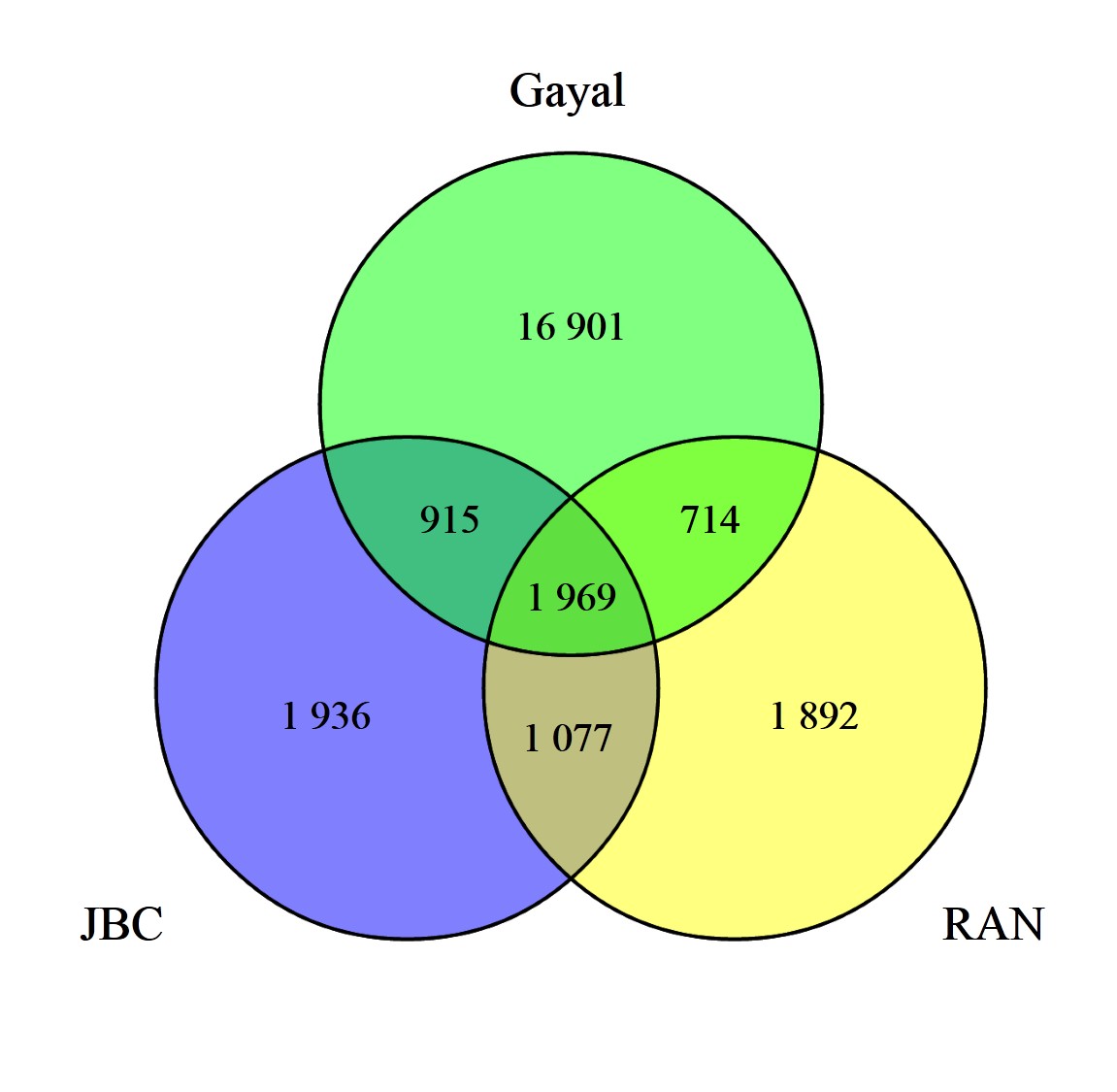


**Figure S3. Venn diagram showing the distribution of shared and breed-specific nonsynonymous SNPs detected in Gayal, RAN and JBC.**


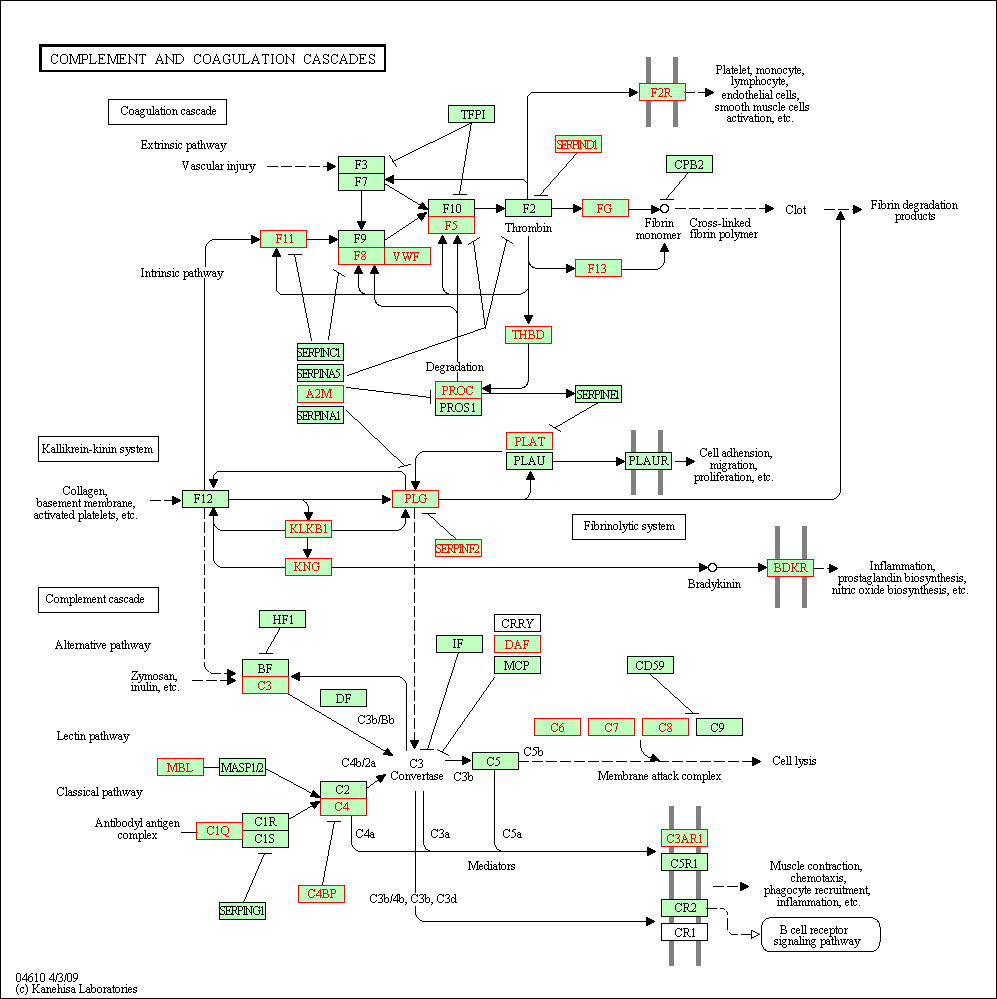


**Figure S4. Pathway of Complement and coagulation cascades.** Significantly enriched genes in the complement and coagulation cascade pathway were marked in red.


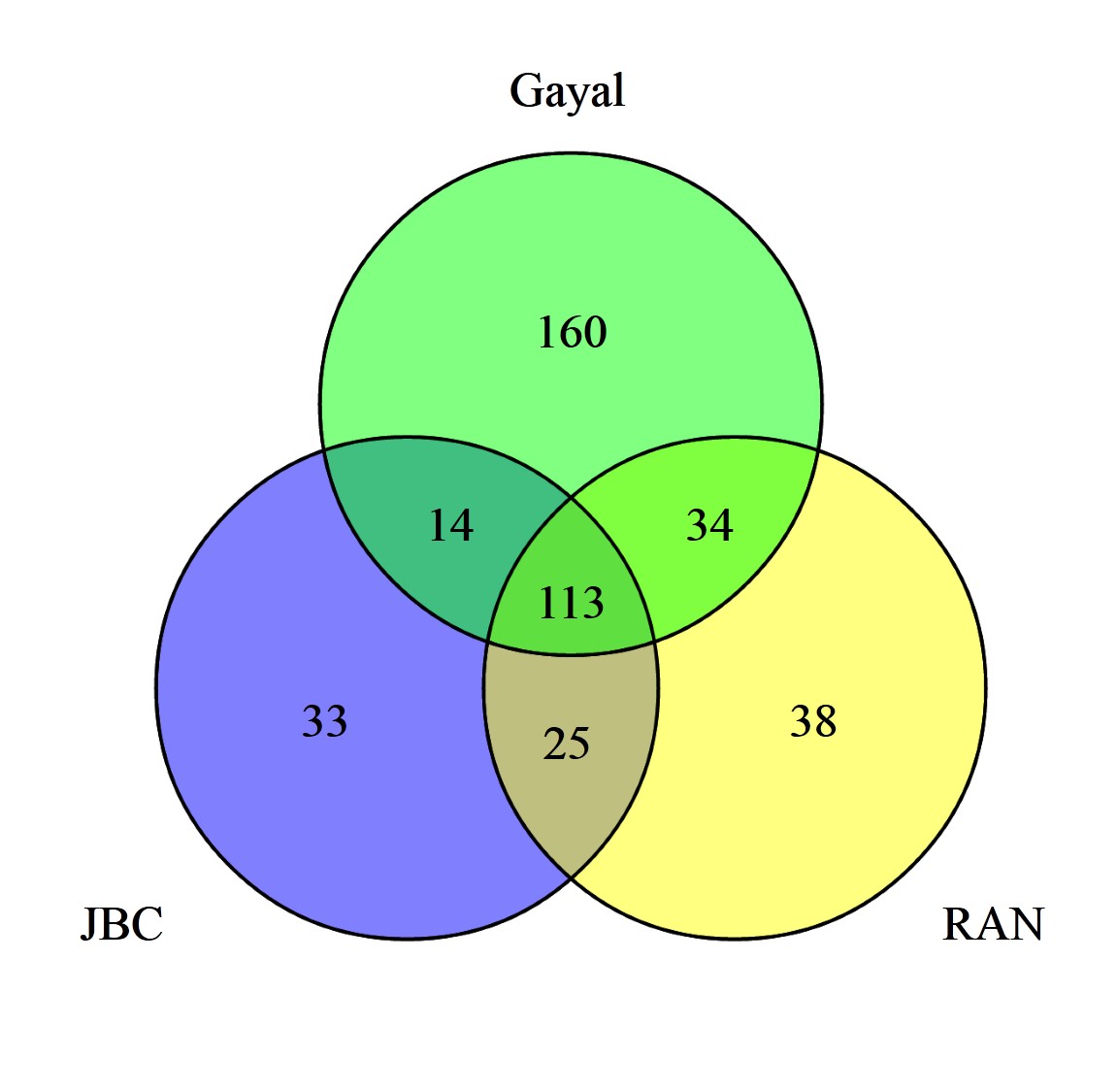


**Figure S5. Venn diagram showing the distribution of shared and breed-specific loss-of-function (LOF) InDels (stop-gains, frameshift InDels in coding sequence and disruptions to essential splice sites) detected in Gayal, RAN and JBC.**


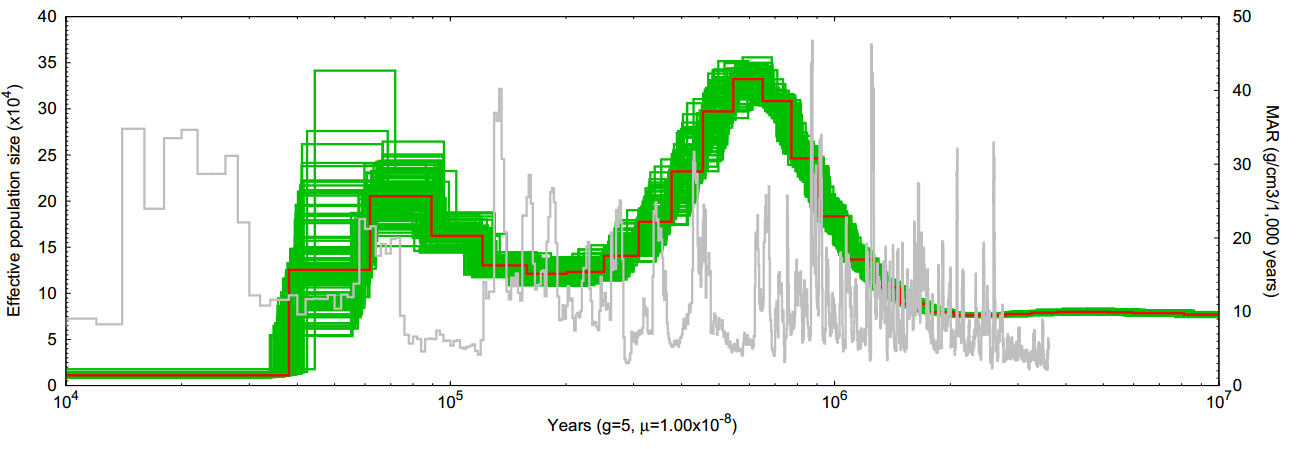


**Figure S6. Demographic history of gayal (*B. frontalis*).** Autosomal SNPs of the sequenced cattle breeds were used to reconstruct demographic history with the pairwise sequentially Markovian coalescent (PSMC) model with the generation time (g = 5), mutation rates (µ = 1×10^−8^).


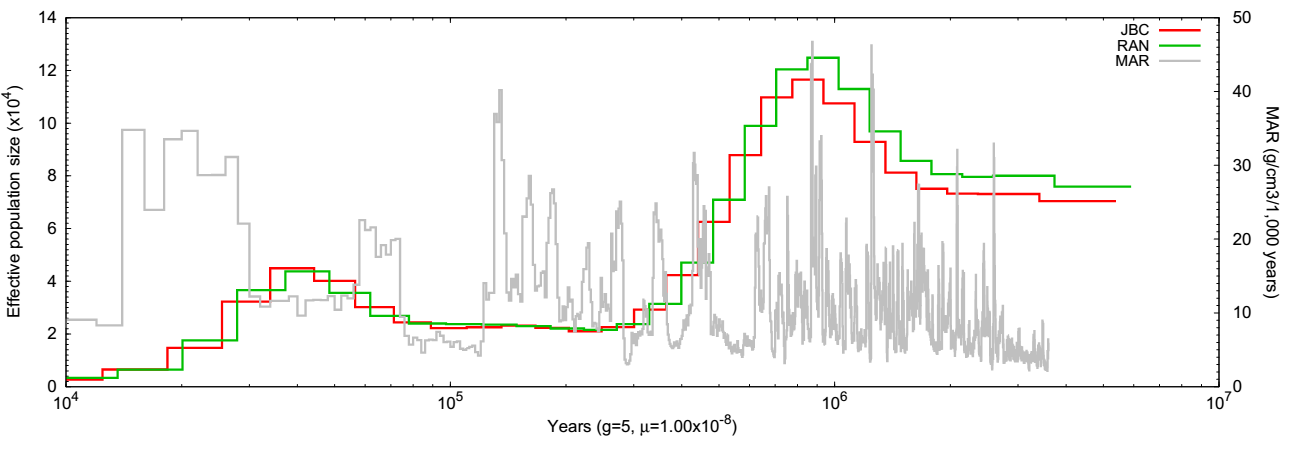


**Figure S7. Demographic history of *Bos taurus*.** Autosomal SNPs of the sequenced cattle breeds were used to reconstruct demographic history with the pairwise sequentially Markovian coalescent (PSMC) model with the generation time (g = 5), mutation rates (µ = 1×10^−8^).

**Supplementary Tables:**

**Table S1.** Information of the sequenced cattle breeds. (XLS)

| **Breed** | **Number** | **Sample Source** | **Status** | **Subspecies** |
| --- | --- | --- | --- | --- |
| Gayal | 1 | Yunnan, Kunming | Semi-wild | *Bos frontalis* |
| Red Angus (RAN) | 1 | Shihezi, Xinjiang | Domestic | *Bos taurus* |
| Japanese Black cattle (JBC) | 1 | Bozhou, Anhui | Domestic | *Bos taurus* |
| Total | 3 | --- | --- | --- |

**Table S2.** Annotation of CNVs and SVs. (XLS)

| Table S2a: Annotation of CNV variants | | | |
| --- | --- | --- | --- |
| CNV varaints | Gayal | RAN | JBC |
| Total numbers | 3,659 | 2,607 | 2,925 |
| Intergenic | 2,681 | 1,851 | 2,101 |
| Upstream | 28 | 9 | 17 |
| Downstream | 12 | 7 | 10 |
| Gene | 938 | 740 | 797 |

Table S2b: Annotation of SV variants

| SV varaints | Gayal | RAN | JBC |
| --- | --- | --- | --- |
| Total numbers | 70,810 | 49,890 | 39,482 |
| Intergenic | 54,644 | 38,891 | 30,655 |
| Upstream | 293 | 238 | 193 |
| Downstream | 263 | 173 | 138 |
| Gene | 15,610 | 10,588 | 8,496 |

**Supplementary Table S3-S10** were submitted as ‘**Supplementary Dataset**’ in the manuscript tracking system:

**Table S3.** Functional analysis of breed-specific nsSNPs and breed-specific nsSNP containing genes in gayal

(XLS)

**Table S4.** Functional analysis of breed-specific InDels and breed-specific InDels containing genes in gayal

(XLS)

**Table S5.** KEGG and GO analysis of breed-specific nsSNP containing genes in gayal.

(XLS)

**Table S6.** Functional analysis of breed-specific nsSNPs and breed-specific nsSNP containing genes in RAN

(XLS)

**Table S7.** Functional analysis of breed-specific InDels and breed-specific InDel containing genes in RAN.

(XLS)

**Table S8.** Functional analysis of breed-specific nsSNPs and breed-specific nsSNP containing genes in JBC.

(XLS)

**Table S9.** Functional analysis of breed-specific InDels and breed-specific InDel containing genes in JBC.

(XLS)

**Table S10.** List of single ortholog copy genes used in phylogenetic analysis.

(XLS)

**Table S11.** List of mitochondrial genomes used in phylogenetic analysis.

(XLS)
